# The Effects of Lipids on α-Synuclein Aggregation In Vitro

**DOI:** 10.3390/biom13101476

**Published:** 2023-10-02

**Authors:** Jennifer Ramirez, Samantha X. Pancoe, Elizabeth Rhoades, E. James Petersson

**Affiliations:** 1 Graduate Group in Biochemistry and Molecular Biophysics, Perelman School of Medicine, University of Pennsylvania, 421 Curie Boulevard, Philadelphia, PA 19104, USA; jennifer.ramirez@pennmedicine.upenn.edu; 2 Department of Chemistry, University of Pennsylvania, 231 South 34th Street, Philadelphia, PA 19104, USA; 3 Department of Biochemistry and Biophysics, Perelman School of Medicine, University of Pennsylvania, 421 Curie Boulevard, Philadelphia, PA 19104, USA

**Keywords:** α-synuclein, lipid, aggregation

## Abstract

The small neuronal protein α-synuclein (αS) is found in pre-synaptic terminals and plays a role in vesicle recycling and neurotransmission. Fibrillar aggregates of αS are the hallmark of Parkinson’s disease and related neurodegenerative disorders. In both health and disease, interactions with lipids influence αS’s structure and function, prompting much study of the effects of lipids on αS aggregation. A comprehensive collection (126 examples) of aggregation rate data for various αS/lipid combinations was presented, including combinations of lipid variations and mutations or post-translational modifications of αS. These data were interpreted in terms of lipid structure to identify general trends. These tabulated data serve as a resource for the community to help in the interpretation of aggregation experiments with lipids and to be potentially used as inputs for computational models of lipid effects on aggregation.

## 1. Introduction

Parkinson’s disease (PD), Parkinson’s disease with dementia (PDD), dementia with Lewy bodies (DLB), and multiple system atrophy (MSA) are considered synucleinopathies: a term that encompasses a group of age-related neurodegenerative diseases featuring aggregation of the protein α-synuclein (αS). PD, the second most prevalent neurodegenerative disease following Alzheimer’s disease, affects approximately 0.3% of the global population and accounts for around 15% of all dementia cases [1]. Usually appearing around age 55, PD is characterized by motor impairments, like resting tremors, rigidity, and bradykinesia, along with possible non-motor symptoms. such as sleep disorders, hallucinations, dementia, autonomic dysfunction, and mood disorders [1,2]. Pathologically, the diagnostic criteria for PD involve the loss of dopaminergic neurons in the substantia nigra, as well as the presence of aggregated αS inclusions within Lewy bodies (LBs) and Lewy neurites (LNs) [3].

**Figure 1:**
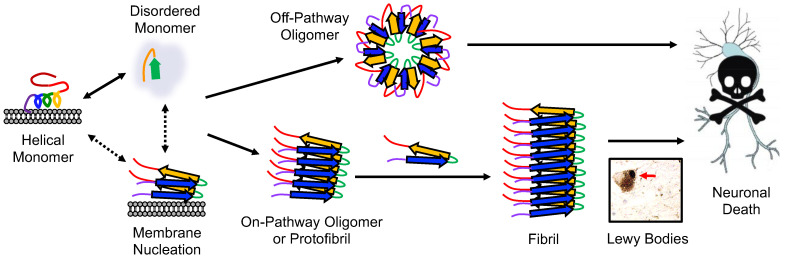
Conformational states and aggregation pathways of αS. Aggregation can either be initiated from free αS or membrane-bound αS. The protein sequence has been colored as in Figure 3.

αS, consisting of 140 amino acids, can be divided into three regions. The N-terminal region (residues 1–90) facilitates αS’s interactions with membranes, and contains seven repeats featuring a conserved KTKEGV motif [4]. The central section, known as the non-amyloid-β component (NAC, residues 61–95), is mainly hydrophobic and plays a role in fibril formation. The C-terminal domain (residues 96–140) is acidic and entirely disordered [4,5]. The exact tertiary structure of wild-type (WT) αS is not universally agreed upon. There are varying propositions regarding the native structure of αS; it can exist as a disordered monomer, but its N-terminus and NAC region adopt a helical conformation when interacting with membranes, while the C-terminus remains unstructured (Figure 1) [6]. Some suggest that αS exists as a stable tetramer that resists aggregation, but disruptions of these tetramers can release monomers prone to misfolding and aggregation [7].

The exact native function of αS remains unclear, but it is mainly found in the presynaptic termini of neurons [8]. αS’s location at presynaptic sites and its interactions with curved membranes suggest a role in synaptic functions, like neurotransmitter release, plasticity [9], and vesicle trafficking [10], and endocytosis [11]. It has also been shown to enhance fusion pore dilation, a dose-dependent effect seen with both endogenous and overexpressed proteins [12]. It interacts with various partners, such as the plasma membrane, synaptic vesicles, soluble N-ethylmaleimide-sensitive factor activating protein receptor (SNARE) protein complexes, proteins involved in dopamine homeostasis, and proteins involved in calcium regulation [13]. These interactions have led to a variety of proposed functions for αS, including apoptosis suppression, modulation of calcium levels through calmodulin, involvement in assembling SNARE complexes and regulating neurotransmitter release, antioxidation, neuronal differentiation, dopamine biosynthesis, and maintenance of polyunsaturated fatty acid levels. It has also been suggested that disruption of αS interactions with membranes may contribute to the mechanism of toxicity in PD [14].

In PD pathology, αS initially forms misfolded monomers or dimers that subsequently assemble into oligomers. These oligomers can further aggregate with monomeric αS to form fibrils, which are the main constituents of LBs (Figure 1) [3]. The aggregation of αS and its interactions with organelles, like mitochondria, autophagosomes, and endo/lysosomes, are thought to be responsible for the toxicity associated with LB formation [15]. Additionally, accumulated αS associates with the inner mitochondrial membrane [16]. Mitochondria are vital for the high energy demand of dopaminergic neurons; thus, αS aggregation can harm the mitochondria, which in turn increases reactive oxygen species (ROS), leading αS to become eliminated via autophagy–lysosome pathways [17]. Fibrils play a crucial role in disease progression by fragmenting and generating seeds that recruit unstructured monomeric αS, leading to the formation of new oligomers [18]. The seeding process can induce aggregation in neighboring neurons, leading to the gradual decline of neuronal function observed in PD [19]. The role and toxicity of oligomers in PD pathology further adds to this complexity, as their diverse range of structures and molecular weights obstructs their assessment [20]. Moreover, the comparison of αS studies is hindered by the utilization of diverse in vitro, cell, and animal models, along with variations in aggregation conditions [21].

There is a growing body of evidence indicating that the aggregation of αS is impacted by lipid membranes [22,23], and in a reciprocal relationship, the presence of bound aggregates can significantly disrupt membrane integrity [24,25,26]. At high lipid/αS ratios, almost all proteins bind to vesicle surfaces without aggregation due to the absence of free αS [27]. At low ratios, free monomers trigger faster primary nucleation on vesicles, significantly surpassing bulk solution rates, likely due to higher protein concentration on vesicles and favorable conformations [28]. Vesicle composition can also impact nucleation, as detailed below.

The αS aggregation process can be separated into primary nucleation, elongation, and secondary nucleation steps. During primary nucleation, monomeric αS assembles into small oligomers known as nuclei, involving a conformational change of disordered αS into a partially structured intermediate that can aggregate [29]. Elongation occurs as monomers join the ends of nuclei, resulting in the formation of longer fibrils. Secondary nucleation involves the creation of new nuclei from pre-existing aggregates called seeds [30]. Fibril fragmentation can also occur so that net fibril amounts ultimately reach a steady state.

**Figure 2:**
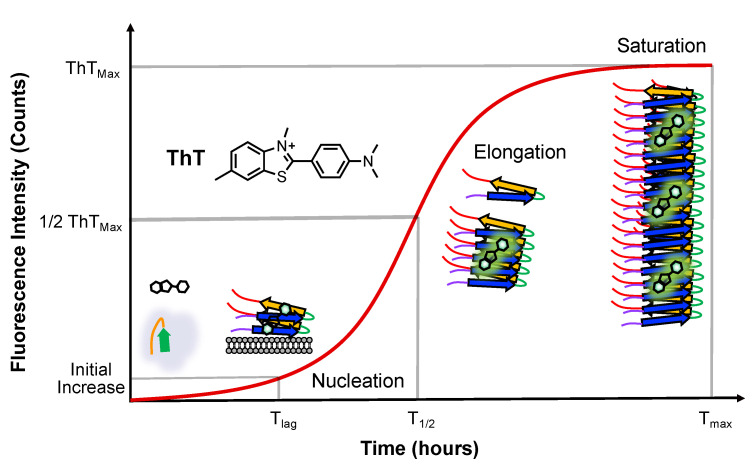
Aggregation monitored via ThT fluorescence. ThT emission changes are shown with major αS conformational states populated at each stage. The timepoints of half maximal ThT fluorescence (T_1/2_) are used to determine the effects of lipids on αS aggregation.

In vitro fibril formation is often used to study αS aggregation. The fibrils are generated by incubating recombinantly expressed human αS, often with agitation, but sometimes under quiescent conditions [31]. Fibril formation and kinetics are assessed using dyes like Thioflavin T (ThT), which undergoes spectral shifts upon binding to amyloid fibrils [32]. ThT aggregation assays involve either periodic sampling or continuous monitoring with a fluorescence plate reader. ThT is widely used due to its ease of implementation, although other techniques, like monitoring via fluorescence polarization of an attached dye, can be used when there is concern that ThT binding may be compromised [33,34]. ThT fluorescence reveals three phases of aggregation: the lag phase (T_lag_), the amount of time required to detect an initial change in fluorescence, the growth phase, exhibiting an exponential signal increase to monomer addition and fibril fragmentation, which includes the T_1/2_, which is defined as the time to reach half of the maximum fluorescence, and the saturation phase, which includes the (T_max_) time to reach maximum fluorescence, when the monomer is depleted and minimal or no net fibril growth occurs (Figure 2).

We note that the lag phase corresponds to primary nucleation, the growth phase encompasses both elongation and secondary nucleation, and the saturation phase includes both fibril fragmentation and secondary nucleation. The rate of fibril formation, determined through fitting the sigmoidal ThT curve, depends on the initial concentrations of monomer and seeds. Fibril seeds can expedite fibril formation through bypassing primary nucleation and reducing the lag time [30]. Factors influencing the rate of αS aggregation encompass mutations, post-translational modifications (PTMs), small molecules, membranes, surfaces, salts, pH, temperature, and agitation, as well as other peptides and proteins [35,36]. Lipid bilayers can significantly accelerate the aggregation kinetics of αS through surface-driven catalysis. This acceleration can exceed three orders of magnitude compared to nucleation in solution [28]. A proposed mechanism for toxicity involves αS oligomers and fibrils assembling at the membrane surface, leading to lipid extraction and eventual membrane disruption [37].

There have been a large number of studies of the effects of mutations and PTMs on αS aggregation, and we have recently summarized these in a comprehensive review [36]. In the context of interpreting mutation and PTM effects, we also discussed the effects of extrinsic factors, including salts, pH, temperature, and agitation. Here, we focused on αS interactions with lipids, which impact the aggregation rate and have important physiological relevance, as the type of lipid that αS interacts with will vary with cell type and subcellular location. While we refer the reader to our recent review for a general overview of the effects of mutations and PTMs, there are a few that should be introduced here, since we discuss them in the context of lipid effects. The SNCA gene responsible for encoding αS is associated with several mutations, including A_30_P, E_46_K, A_53_T, H_50_Q, and G_51_D, which are linked to inherited forms of PD [38,39,40,41,42,43,44,45]. These familial mutants of αS exhibit distinct biophysical properties, such as differences in the aggregation rate, lipid binding, and fibril structure, when compared to the WT αS [46,47,48]. For example, in the rat optic system, αS is transported by axonal vesicles via its amino-terminal repeat region. The A_30_P mutation disrupts this vesicle-binding activity, while A_53_T does not [49]. While another study indicated that A_30_P and A_53_T have been shown to display limited impact on lipid binding [50], the majority of subsequent studies have demonstrated that A_30_P binds more weakly to lipids [51,52,53]. Another comprehensive in vitro analysis revealed significantly reduced lipid binding for A_30_P, moderately diminished binding for G_51_D, and only slight reductions in binding for the other mutants [54]. Furthermore, the more rapidly aggregating αS variant dictated the lag time in mixed preparations of A_30_P or A_53_T and WT αS [54]. There is substantial evidence suggesting that various PTMs of αS significantly influence αS’s aggregation ability [55,56]. For example, phosphorylation at serine 129 is a defining hallmark of PD and promotes aggregation [57], while SUMOylation impairs αS ubiquitination and prevents αS degradation, promoting its aggregation [58]. Two PTMs that are relevant for this review are N-terminal acetylation (AcN), which increases membrane binding and is a common feature of mammalian αS [59], and 4-hydroxy-2-nonenal (HNE) covalent adducts, which primarily form under oxidizing conditions with His 50. 

**Figure 3:**
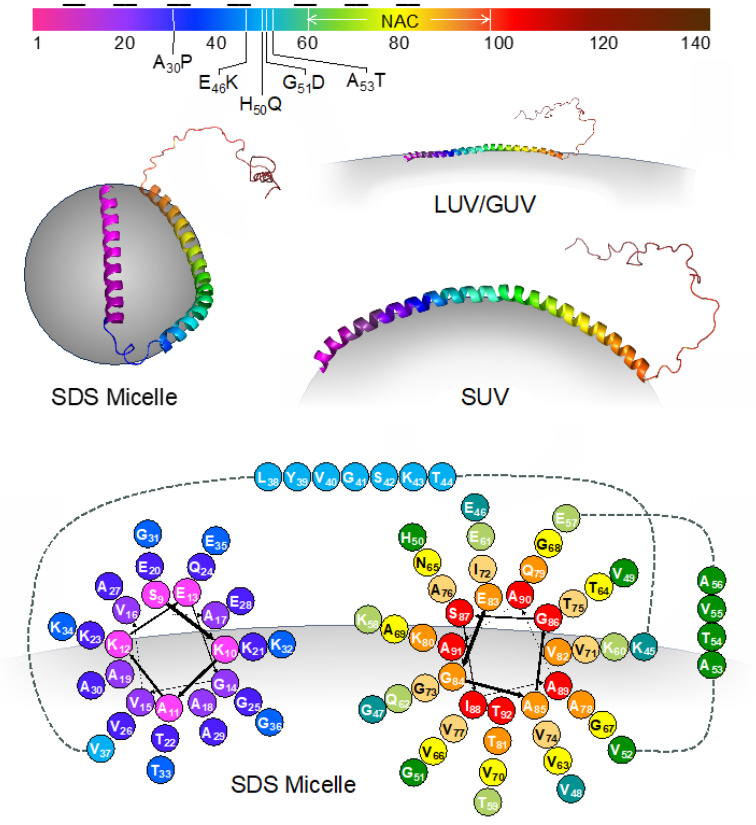
Membrane-bound conformations of αS. Top: The sequence of αS colored according to residue number as in structures and cartoons throughout the article. The imperfect repeat segments are indicated by black horizontal lines. The positions of discussed mutations and the NAC region are also shown. Middle left: the structure of αS bound to an SDS micelle (PDB ID 1qx8). Middle right: models of αS bound to SUVs or LUVs/GUVs illustrating the effect of membrane curvature on helical conformation. Bottom: helical wheel diagram showing amino acid positioning relative to the SDS micelle surface for residues 9–92.

Vesicle binding causes a conformational change, wherein the N-terminal and NAC regions adopt a helical structure that may or may not include a bend depending on vesicle curvature and composition (Figure 3) [60,61]. Studies have also suggested that the NAC domain may promote organization on the lipid bilayer surface through the stabilization of protein–lipid complexes [62]. The biophysical impacts of membrane binding encompass increased affinity for highly curved bilayers [61], hindrance of small vesicle fusion [63], and the alteration of large, flat membranes through tubulation and vesiculation [64]. For instance, the V_70_P mutation has been identified as preventing αS from forming a bridge between the vesicle and plasma membranes [65,66]. This bridge formation, facilitated via a specific broken helix configuration, has been hypothesized to be necessary for its inhibitory function [67].

Conformational changes in αS may lead to different aggregation kinetics. In order to study these interactions, liposomes with different head groups and fatty acid chains have been synthesized. Lipid vesicles are categorized based on their lamellarity, such as unilamellar vesicles (UVs). Additionally, UVs can be further classified into small unilamellar vesicles (SUVs), with a particle size below 50 nm, large unilamellar vesicles (LUVs), with a particle size ranging from 50 nm to 1000 nm (although experimental methods typically limit preparations to >75 nm), and giant unilamellar vesicles (GUVs), with a particle size exceeding 1 μm [68]. SUVs are used as models of secretory vesicles, which are important for studying αS’s role in SNARE complex assembly. LUVs have been used to demonstrate fluorescence correlation spectroscopy’s effectiveness in quantifying αS binding to lipid vesicles [69]. GUVs can serve as models of cellular or organelle membranes. While the N-terminus and NAC regions of αS have been shown to adopt a helical conformation upon interaction with all forms of membranes, the curvature of and number of helical segments depend on the curvature of the membrane [61]. For example, upon binding to SDS micelles, αS adopts a broken helix conformation, structurally characterized via NMR [6]. While atomic coordinates have not been reported for the other forms of membrane-bound αS, it is clear that the 1–100 region adopts a single helix, and the positioning of residues relative to the membrane has been determined for some vesicle sizes [66]. A helical wheel diagram illustrating amino acid positioning for the SDS micelle form of αS is shown in Figure 3, and the general pattern of the positions of positively charged, negatively charged, and hydrophobic amino acids is preserved when αS is bound to other types of membranes.

Specific lipid types have also been shown to influence how monomeric αS associates with membranes [70]. The positively charged nature of the N-terminal domain enables it to engage in electrostatic interactions with negatively charged membrane lipids [51], which are notably abundant in synaptic vesicle membranes [71]. This domain exhibits an inclination towards glycosphingolipids, and specifically, residues 34–45 have been suggested to constitute a lipid-binding motif on the cell surface, featuring an exposed aromatic residue [72]. Additionally, truncation of the C-terminus of αS has been shown to increase its interaction with the phospholipid cardiolipin, which is unique to mitochondria [73]. Lipid size, lipid composition, and the ratio of lipids to proteins (L:P) have been shown to have an impact on αS aggregation kinetics [4,54,74,75].

**Figure 4:**
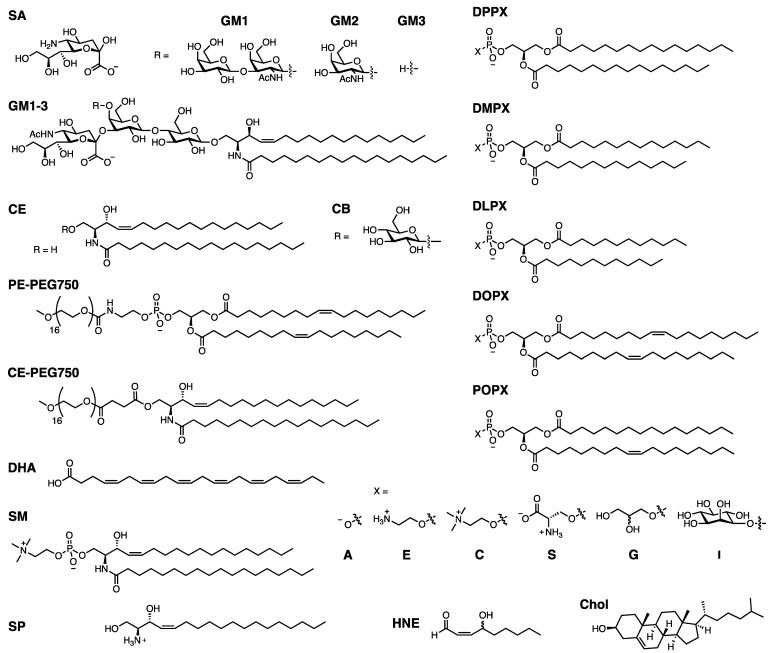
Lipid structures. General structures are given for the DPPX, DMPX, DLPX, DOPX, and POPX lipids, where X = A, E, C, S, or G, or I.

Since we will discuss a variety of lipids which are typically referred to by abbreviated names, the full names and abbreviations are listed in Table 1 below, and the structures and abbreviated names are shown in Figure 4 for convenient reference. Note: some abbreviations are not mentioned in the text and only appear in Table 2 and Table 3.

## 2. Collected Aggregation Studies

### 2.1. Summary of Studies

Lipids have been found in LBs [76], and lipids and αS have been shown to co-aggregate in vitro [77,78,79], underscoring the importance of the interactions between lipids and αS. αS is thought to interact with a number of biological membranes, causing changes that may explain some of the cellular dysfunction associated with αS. αS has also been shown to cause membrane remodeling by changing membrane curvature [80,81]. Toxic αS oligomers can permeabilize membranes [82]. Together, these toxic functions of αS are thought to play a role in mitochondrial dysfunction and impaired trafficking [80]. In the context of other modifiers of aggregation, exposure of αS to lipids can result in lipid peroxidation products like HNE. Additionally, all of the familial PD mutations occur in the region of αS that is thought to interact with lipids. The impact of lipid binding on αS is variable, with reports of both increased and decreased aggregation rates. Properties such as chain length [83], head group [78,84], and charge [85] vary among lipids, and have been shown to impact αS aggregation. Additionally, the actual composition of membranes varies widely based on the organelle or vesicle, impacting membrane curvature and fluidity [86]. As αS is involved in vesicle trafficking, most studies aim to replicate vesicles. Lipids in synaptic vesicles are made of Chol, phospholipids (POPC, 32%; POPS, 12%; POPE, 54%; POPI, 2%), and SM (7%) [74]. Synaptic vesicles are commonly mimicked by vesicles formulated from 50:30:20 DOPE/DOPS/DOPC [75,87,88]; however, most studies tend to solely focus on one headgroup, potentially limiting their applicability. Finally, it should be noted that physical aggregation conditions also play a role in determining the rate of aggregation. Compared to other classes of modifiers of αS, there are a large number of studies on lipids which assess aggregation under quiescent conditions. Since agitation is thought to play a large role in secondary nucleation and aggregation rates overall, this difference makes it difficult to compare findings across studies. Table 2 and Table 3 below summarize how the aggregation rates with lipids and/or mutation to αS are scored through qualitatively comparing the relative rate to the corresponding WT control.

### 2.2. Phospholipids

For phospholipids, a major factor in their impact on aggregation is the L:P ratio during incubation and fibril formation [74]. Each type of lipid can be characterized by the number of lipid molecules that interact with one molecule of αS. When the L:P ratio is less than this number, there will be αS that is bound to lipids and αS that is free in solution. Both of these populations are thought to be necessary for fibril formation, with the bound αS potentially serving as a nucleation site and the free monomer allowing for elongation [28,78,89,90]. This condition allows for increased aggregation rates induced via lipids. However, when the L:P ratio is greater than the number of lipid molecules bound to αS, then all αS is thought to be bound to lipids. The helical form adopted by membrane-bound αS has been thought to reduce aggregation, as αS is unable to adopt the partially folded conformation needed to form fibrils [91]. Additionally, without free monomers, fibrils cannot form, and aggregation is inhibited [28,74].

To characterize the negatively charged PS headgroup, a number of studies have compared lipids with different chain lengths and saturations with PS headgroups. Galvagnion et al. found that by changing the concentration of lipids, DMPS lipids accelerate aggregation up to a L:P ratio of 15, after which they reduced the rate of acceleration. They also found that increasing the concentration of protein led to an increased rate of aggregation when incubating αS for both DMPS SUVs and LUVs [28]. To compare the effect of lipid length on aggregation, they incubated DMPS or DLPS lipids with increasing concentrations of αS, finding that for a given lipid concentration, increasing the concentration of protein accelerated fibril formation, and that the shorter DLPS lipids increased the rate of aggregation more than the longer DMPS lipids. Similarly, they saw that holding the concentration of protein constant and increasing the concentration of DLPS led to increased rates of aggregation. They also studied longer lipids, like DOPS, POPS, and DPPS, at 1:1 L:P ratios, finding that none of these lipids were able to induce aggregation. Since the length of the hydrocarbon chain is strongly correlated with a lipid’s solubility, changes in lipid solubility may govern how they modulate aggregation [75]. Similarly, Hoover et al. found that at a 10:1 L:P ratio, αS aggregated fastest with DLPS SUVs, then DMPS, and finally DOPS, confirming that the rate of aggregation is inversely proportional to the length of the acyl chain [92]. The aggregation of αS with DOPS lipids likely depends on the L:P ratio, with Galvignon et al. finding that a 1:1 ratio did not aggregate [75], and Mahapatra et al. finding that incubation with DOPS vesicles at a L:P ratio of 10:1 increased the rate of aggregation compared to that of the WT. In the context of familial mutants, DMPS was shown to increase the rate of aggregation for A_53_T, to slightly reduce the rate of aggregation of A_30_P, and to greatly reduce the rates of aggregation for H_50_Q, E_46_K, and G_51_D. For all mutants which aggregated, increasing the concentration of protein increased their rate of aggregation [89].

Vesicles made from lipids with negatively charged PG headgroups seem to inhibit aggregation. At a L:P ratio of 20:1, DOPG vesicles accelerate aggregation, while at a L:P ratio of 50:1, they inhibit aggregation [93]. POPG follows a similar concentration dependence, with a L:P ration of 1:1 stimulating aggregation and a L:P ratio of 50:1 greatly reducing the rate of aggregation [78,79]. Interestingly, POPG vesicles were shown to co-aggregate with αS [78]. In contrast, DPPG SUVs at 1:1, 4:1, and 12:1 L:P ratios did not cause significant changes in the rate of aggregation. This difference may be due to the length of the acyl chain or the aggregation conditions [23].

PC forms zwitterionic lipids and is also the most abundant headgroup in animal cells [83]. Zhu et al. found that DPPC vesicles are somewhat inhibitory, with increasing L:P ratios leading to decreased rates of aggregation [91]. Similarly, Jiang et al. found that POPC, which is able to be remodeled via αS, was able to inhibit fibril formation in a concentration-dependent manner. They also tested vesicles made from 1:1 POPC/POPA, which is not remodeled by αS, at the same concentrations and found that these vesicles slightly accelerate aggregation. They hypothesize that lipids which are able to be remodeled are likely to inhibit aggregation due to some of the αS being involved in remodeling, lowering the effective concentration of free αS that can form fibrils [90]. O’Leary et al. found that both DOPC and DPPC do not have an effect on AcN αS at a L:P ratio of 1:1. At a higher ratio of 10:1, DOPC vesicles inhibit fibril formation, while DPPC vesicles do not. Since DOPC is a fluid-phase membrane and DPPC is a gel-phase membrane, they proposed that membrane fluidity may play a role in aggregation kinetics, with gel-phase membranes immobilizing more protein and allowing for enhanced primary nucleation through interaction with free monomers [83]. In contrast, Zhu et al. found that DPPC SUVs at 1:5, 1:1, and 5:1 ratios did not impact the rate of aggregation [85].

Other groups saw reduced aggregation via DOPC SUVs at a 20:3(124) ratio and inhibited aggregation at a range of L:P ratios [94]. Kurochka et al. saw that 3:1 POPC/POPG SUVs at ratios of 1:8, 1:4, 1:2, 1:1, 2:1, 4:1, 8:1, and 18:1 reduced the rate of aggregation, and that increased amounts of lipid led to increased lag times. They also examined 4:1 POPC/POPG SUVs, which are slightly less negatively charged, and saw that the addition of lipids increased the lag time relative to that of the WT, although there was no consistent correlation between the lag time and the amount of lipid present [23].

Zhu et al. also tested a number of vesicles with 1:1 mixtures of phospholipids with different head groups, all of which had a net charge of −1. Compared to zwitterionic DPPC vesicles, these negatively charged vesicles were able to significantly impact the rate of aggregation. Looking at L:P ratios of both DPPA/DPPC and DPPG/DPPC SUVs and LUVs, they found that 1:5 L:P ratios accelerated fibril formation, while 5:1 L:P ratios decreased the rate of fibril formation. However, the extent to which fibril formation was slowed was dependent on the size of the vesicles. DPPA/DPPC and DPPG/DPPC LUVs slowed the rate of fibril formation, while DPPA/DPPC and DPPG/DPPC SUVs nearly inhibited fibril formation. This is likely because αS binds weakly to LUVs as compared to SUVs, meaning that the effective concentration of free αS is higher in the case of LUVs. Moreover, 1:1 L:P ratios of DPPC/DPPA SUVs and LUVs, along with DPPG/DPPC SUVs, did not impact the rate of aggregation, while a 1:1 ratio of DPPG/DPPC LUVs was shown to slightly reduce the rate of aggregation [85,91]. DPPA/DPPE and DPPG/DPPE vesicles also showed increased rates of aggregation at 1:5 ratios and inhibited aggregation at 5:1 ratios. At 1:1 ratios, DPPA/DPPE and DPPG/DPPE vesicles showed slower aggregation. The PS headgroup may be particularly inhibitory, with complete inhibition of aggregation for DPPS/DPPC vesicles at 1:5 and 10:1 ratios, and DPPS/DPPE vesicles at a 10:1 ratio. At a 1:2 ratio, however, DPPS/DPPC SUVs were able to accelerate fibril formation [85].

### 2.3. Gangliosides

Gangliosides are a class of glycosphingolipids that have a SA group attached to an oligosaccharide headgroup. They are primarily found in the outer plasma membrane and in exosomes [94]. Martinez et al. found that when incubated at a 10:1 ratio with αS, vesicles made from 1:1 DPPC to cerebrosides or ceramides accelerated the rate of fibril formation, while DPPC/total ganglioside vesicles slowed aggregation, and DPPC/GM1 vesicles inhibited aggregation. Both DPPC/GM2 and DPPC/GM3 vesicles, whose headgroups differ from GM1 in the number and type of sugars in the headgroup, did not aggregate. DPPC/GM1 vesicles also inhibited the aggregation-prone mutant A_53_T αS [84]. Similarly, Bartels et al. found that a 10:1 ratio of GM1 to αS reduced the rate of aggregation for WT αS and inhibits fibril formation for N-terminally acetylated αS [95]. In contrast, Gaspar et al. found that gangliosides accelerate fibril formation relative to WT, with a greater L:P ratio leading to faster rates. Specifically, 9:1 DOPC/GM1 and 9:1 DOPC/GM3 accelerated fibril formation more than 9:1 DOPC/PE-PEG750, a synthetic analogue that is similar in size and charge to GM1. They also studied DOPC/asialo-GM1, which is zwitterionic and lacks SA in the GM1 headgroup, and DOPC/CE-PEG750, another zwitterionic headgroup. Both of these headgroups can aggregate, although their aggregation is severely impaired when compared to lipids with negatively charged headgroups [94]. The difference in these results may be due to aggregation conditions, as both Martinez and Bartels agitated the αS protein solution, while Gaspar performed the ThT assay under quiescent conditions. Additionally, Gaspar conducted the ThT assay at a slightly acidic pH (5.5) to mimic the conditions in lysosomes and endosomes. An acidic pH is known to accelerate fibril formation [96].

### 2.4. Polyunsaturated Fatty Acids

The concentration and composition of polyunsaturated fatty acids changes with age, coinciding with an increase in lipid peroxidation. Specifically, DHA is present at high levels in brain areas that also have LBs and LNs in PD patients [97]. DHA is thought to accelerate fibril formation at a ratio of 10:1 [98], and to nearly inhibit aggregation at a ratio of 50:1 [93,98]. At 50:1, the oligomers that do form are off-pathway and contain both αS and DHA. Additionally, since DHA is involved in lipid peroxidation, αS that is aggregated in the presence of DHA is subjected to PTMs, such as Met oxidation, and the addition of reactive peroxidation byproducts, both of which may have an impact on aggregation rates [98].

### 2.5. Biological Membrane Mimics

Since one of αS’s proposed functions is aiding vesicle trafficking and SNARE complex assembly, the interaction of αS with biologically relevant membranes, such as lipid rafts, vesicles, and exosomes, is important. Synthetic vesicles composed of 2:2:1 DOPC/SM/Chol are used as mimics of lipid rafts. When incubated with N-terminally acetylated αS at a 1:1 or 10:1 L:P ratio, the aggregation rate is unchanged [83]. Multiple studies have used 5:3:2 DOPE/DOPS/DOPC as a model for synaptic vesicles. Sinha et al. also found that increasing concentrations of 5:3:2 DOPE/DOPS/DOPC slowed aggregation; however, they used HNE-modified αS [99]. Mahapatra et al. found that at a ratio of 10:1, these vesicles accelerate fibril formation [88]. As a follow up, they investigated the impact of Chol on lipid binding due to its role in modulating αS binding to lipid rafts. They used SUVs containing 5:3:2 DOPE/DOPS/DOPC vesicles with 0%, 5%, 10%, 20%, and 40% cholesterol at a ratio of 10:1 L:P. They saw that lag times increased with up to 10% Chol and then decreased. This trend was consistent with the extent of lipid binding. At 10% Chol, 90% of the αS was bound to the vesicles, meaning that there was very little free αS available to aggregate [100].

Grey et al. examined the impact of exosomes from mouse neuroblastoma cells on aggregation, finding that exosomes from cells overexpressing WT αS, WT cells, and cells expressing mutant A_53_T, A_30_P, or E_46_K αS were all able to accelerate fibril formation. Based on the lipid composition of these cells, they created a number of mimics. They found that vesicles made from DOPC alone, DOPC with 6% Chol, or DOPC with 15% SP reduced the rate of aggregation, while vesicles made from DOPC and 10% GM1 or GM3 were able to accelerate aggregation. However, the impact of GM1 and GM3 vesicles on aggregation was concentration-dependent, while the exosomes accelerated fibril formation at all L:P ratios except the highest ratio, indicating that there are still some limitations associated with in vitro vesicles as models of biologically relevant vesicles [101].

**Table 2:** Summary of phospholipid effects on αS aggregation.

Ref.	Rate ^	αS	Lipid	Ratio	Aggregation Conditions
[75]	++	WT	DMPS	5:1, 5:2, 5:3, 5:4, 1:1	20 mM sodium phosphate, pH 6.5, no shaking
[75]	++	WT	DLPS	5:1, 5:2, 5:3, 5:4, 1:1	20 mM sodium phosphate, pH 6.5, no shaking
[75]	**	WT	DLPS	1:2, 1:1, 3:2, 2:1, 5:1, 10:1, 15:1, 20:1	20 mM sodium phosphate, pH 6.5, 50 μM αS, no shaking
[75]	−2	WT	DOPS	1:1	20 mM sodium phosphate, pH 6.5, 100 um α-Syn, no shaking
[75]	−2	WT	POPS	1:1	20 mM sodium phosphate, pH 6.5, 100 μM αS, no shaking
[75]	−2	WT	DPPS	1:1	20 mM sodium phosphate, pH 6.5, 100 μM αS, no shaking
[89]	++	WT	DMPS	5:1, 5:2, 5:3, 5:4, 1:1	20 mM sodium phosphate, pH 6.5, no shaking
[89]	1	A_53_T	DMPS	5:1, 5:2, 5:3, 5:4, 1:1	20 mM sodium phosphate, pH 6.5, no shaking
[89]	−1	A_30_P	DMPS	5:1, 5:2, 5:3, 5:4, 1:1	20 mM sodium phosphate, pH 6.5, no shaking
[89]	−2	E_46_K	DMPS	5:1, 5:2, 5:3, 5:4, 1:1	20 mM sodium phosphate, pH 6.5, no shaking
[89]	−2	H_50_Q	DMPS	5:1, 5:2, 5:3, 5:4, 1:1	20 mM sodium phosphate, pH 6.5, no shaking
[89]	−2	G_51_D	DMPS	5:1, 5:2, 5:3, 5:4, 1:1	20 mM sodium phosphate, pH 6.5, no shaking
[91]	1	WT	DPPC/DPPA SUVs	1:5	20 mM Tris, 100 mM NaCl, pH 7.5, 35 μM αS, shaking
[91]	0	WT	DPPC/DPPA SUVs	1:1	20 mM Tris, 100 mM NaCl, pH 7.5, 35 μM αS, shaking
[91]	−2	WT	DPPC/DPPA SUVs	5:1	20 mM Tris, 100 mM NaCl, pH 7.5, 35 μM αS, shaking
[91]	1	WT	DPPC/DPPA LUVs	1:5	20 mM Tris, 100 mM NaCl, pH 7.5, 35 μM αS, shaking
[91]	0	WT	DPPC/DPPA LUVs	1:1	20 mM Tris, 100 mM NaCl, pH 7.5, 35 μM αS, shaking
[91]	−1	WT	DPPC/DPPA LUVs	5:1	20 mM Tris, 100 mM NaCl, pH 7.5, 35 μM αS, shaking
[91]	**	WT	DPPC	5:1, 10:1, 20:1	20 mM Tris, 100 mM NaCl, pH 7.5, 35 μM αS, shaking
[28]	##	WT	DMPS SUVs	2:1, 4:1, 6:1, 8:1, 10:1, 15:1, 20:1, 30:1, 40:1	20 mM sodium phosphate, 0.01% sodium azide, pH 6.5, 50 μM αS, no shaking
[28]	**	WT	DMPS SUVs	10:1, 10:2, 10:4, 10:6, 10:8, 1:1	20 mM sodium phosphate, 0.01% sodium azide, pH 6.5, no shaking
[28]	**	WT	DMPS LUVs	10:1, 10:2, 10:4, 10:6, 10:8, 1:1	20 mM sodium phosphate, 0.01% sodium azide, pH 6.5, no shaking
[85]	1	WT	DPPG/DPPC 1:1 SUVs	1:5	20 mM Tris-HCl, 100 mM NaCl, pH 7.5, 35 μM αS, no shaking
[85]	0	WT	DPPG/DPPC 1:1 SUVs	1:1	20 mM Tris-HCl, 100 mM NaCl, pH 7.5, 35 μM αS, no shaking
[85]	−2	WT	DPPG/DPPC 1:1 SUVs	5:1	20 mM Tris-HCl, 100 mM NaCl, pH 7.5, 35 μM αS, no shaking
[85]	1	WT	DPPA/DPPC 1:1 SUVs	1:5	20 mM Tris-HCl, 100 mM NaCl, pH 7.5, 35 μM αS, no shaking
[85]	0	WT	DPPA/DPPC 1:1 SUVs	1:5	20 mM Tris-HCl, 100 mM NaCl, pH 7.5, 35 μM αS, no shaking
[85]	−2	WT	DPPA/DPPC 1:1 SUVs	5:1	20 mM Tris-HCl, 100 mM NaCl, pH 7.5, 35 μM αS, no shaking
[85]	1	WT	DPPA/DPPE 1:1 SUVs	1:5	20 mM Tris-HCl, 100 mM NaCl, pH 7.5, 35 μM αS, no shaking
[85]	−1	WT	DPPA/DPPE 1:1 SUVs	1:1	20 mM Tris-HCl, 100 mM NaCl, pH 7.5, 35 μM αS, no shaking
[85]	−2	WT	DPPA/DPPE 1:1 SUVs	5:1	20 mM Tris-HCl, 100 mM NaCl, pH 7.5, 35 μM αS, no shaking
[85]	1	WT	DPPG/DPPE 1:1 SUVs	1:5	20 mM Tris-HCl, 100 mM NaCl, pH 7.5, 35 μM αS, no shaking
[85]	−1	WT	DPPG/DPPE 1:1 SUVs	1:1	20 mM Tris-HCl, 100 mM NaCl, pH 7.5, 35 μM αS, no shaking
[85]	−2	WT	DPPG/DPPE 1:1 SUVs	5:1	20 mM Tris-HCl, 100 mM NaCl, pH 7.5, 35 μM αS, no shaking
[85]	1	WT	DPPG/DPPC 1:1 LUVs	1:5	20 mM Tris-HCl, 100 mM NaCl, pH 7.5, 35 μM αS, no shaking
[85]	−1	WT	DPPG/DPPC 1:1 LUVs	1:1	20 mM Tris-HCl, 100 mM NaCl, pH 7.5, 35 μM αS, no shaking
[85]	−1	WT	DPPG/DPPC 1:1 LUVs	5:1	20 mM Tris-HCl, 100 mM NaCl, pH 7.5, 35 μM αS, no shaking
[85]	0	WT	DPPC SUVs	1:5	20 mM Tris-HCl, 100 mM NaCl, pH 7.5, 35 μM αS, no shaking
[85]	0	WT	DPPC SUVs	1:1	20 mM Tris-HCl, 100 mM NaCl, pH 7.5, 35 μM αS, no shaking
[85]	0	WT	DPPC SUVs	5:1	20 mM Tris-HCl, 100 mM NaCl, pH 7.5, 35 μM αS, no shaking
[85]	1	WT	DPPS/DPPC 1:1 SUVs	1:2	20 mM Tris-HCl, 100 mM NaCl, pH 7.5, 35 μM αS, no shaking
[85]	−2	WT	DPPS/DPPC 1:1 SUVs	1:5	20 mM Tris-HCl, 100 mM NaCl, pH 7.5, 35 μM αS, no shaking
[85]	−2	WT	DPPS/DPPC 1:1 SUVs	10:1	20 mM Tris-HCl, 100 mM NaCl, pH 7.5, 35 μM αS, no shaking
[85]	−1	WT	DPPG/DPPE 1:1 SUVs	10:1	20 mM Tris-HCl, 100 mM NaCl, pH 7.5, 35 μM αS, no shaking
[85]	−2	WT	DPPS/DPPE 1:1 SUVs	10:1	20 mM Tris-HCl, 100 mM NaCl, pH 7.5, μM αS, no shaking
[85]	−2	WT	DPPS/DPPC 1:1 SUVs	10:1	20 mM Tris-HCl, 100 mM NaCl, pH 7.5, 35 μM αS, no shaking
[101]	−1	WT	DOPC SUVs	20:3	10 mM MES, 140 mM NaCl, pH 5.5, 30 μM αS, 100 rpm
[93]	1	WT	DOPG SUVs	20:1	20 mM Tris, 150 mM NaCl, pH 7.4, 50 μM αS, 500 rpm
[93]	−2	WT	DOPG SUVs	50:1	20 mM Tris, 150 mM NaCl, pH 7.4, 50 μM αS, 500 rpm
[83]	0	AcN	DPPC SUVs	1:1	20 mM MOPS, 100 mM NaCl, pH 7, 50 μM αS, shaking
[83]	0	AcN	DPPC SUVs	10:1	20 mM MOPS, 100 mM NaCl, pH 7, 50 μM αS, shaking
[83]	0	AcN	DOPC SUVs	1:1	20 mM MOPS, 100 mM NaCl, pH 7, 50 μM αS, shaking
[83]	−1	AcN	DOPC SUVs	10:1	20 mM MOPS, 100 mM NaCl, pH 7, 50 μM αS, shaking
[88]	1	WT	DOPS	10:1	20 mM sodium phosphate, 1 mM sodium azide, pH 7.4, 5 μM αS, 180 rpm
[79]	−1	WT	POPG	50:1	1:1 PBS:HEPES, pH 7.4, 70 μM αS, 300 rpm
[78]	1	WT	POPG	1:1	20 mM MOPS, 100 mM NaCl, pH 7, 70 μM αS, 600 rpm
[78]	−2	WT	POPG	50:1	20 mM MOPS, 100 mM NaCl, pH 7, 70 μM αS, 600 rpm
[90]	0	WT	POPC	1:1	20 mM MOPS, 100 mM NaCl, pH 7, 70 μM αS, shaking
[90]	−1	WT	POPC	5:1	20 mM MOPS, 100 mM NaCl, pH 7, 70 μM αS, shaking
[90]	−2	WT	POPC	10:1	20 mM MOPS, 100 mM NaCl, pH 7, 70 μM αS, shaking
[90]	1	WT	POPC/POPA 1:1	1:1, 5:1, 10:1	20 mM MOPS, 100 mM NaCl, pH 7, 70 μM αS, shaking
[90]	−2	WT	DOPC	5:1, 10:1, 15:1, 25:1, 50:1	10 mM MES, pH 5.5, 20 μM αS, no shaking

## Changing concentration of lipid; increase up to 15:1; decreases past 15:1. ** For a given protein concentration, the rate increases as the concentration of lipid increases. ++ For a given lipid concentration, the rate increases as the concentration of protein increases. ^ Aggregation rates for mutants, T_1/2_(Mut). Mutants are scored by qualitatively comparing the relative rate, T_1/2_(Mut), to the corresponding WT control, T_1/2_(WT), as follows: +1 for T_1/2_(Mut) < 0.5 T_1/2_(WT), 0 for 0.5 T_1/2_(WT) ≤ T_1/2_(Mut) ≤ 2 T_1/2_ (WT), −1 for 2 T_1/2_ (WT) < T_1/2_ (Mut) < 4 T_1/2_ (WT), −2 for T_1/2_ (Mut) > 2 T_1/2_ (WT) or very shallow, non-sigmoidal aggregation curve.

**Table 3:** Summary of other lipids’ effects on αS aggregation.

Ref.	Rate ^	αS	Lipid	Ratio	Aggregation Conditions
[84]	1	WT	DPPC/CB 1:1	10:1	20 mM Tris-HCl, pH 7.5, 56 μM αS
[84]	1	WT	DPPC/CE 1:1	10:1	20 mM Tris-HCl, pH 7.5, 56 μM αS
[84]	−1	WT	DPPC/GM1-3 1:1	10:1	20 mM Tris-HCl, pH 7.5, 56 μM αS
[84]	−2	WT	DPPC/GM1 1:1	10:1	20 mM Tris-HCl, pH 7.5, 56 μM αS
[84]	−2	WT	DPPC/GM1 1:1	10:1	20 mM Tris-HCl, pH 7.5, 56 μM αS
[84]	−2	WT	DPPC/GM2	10:1	20 mM Tris-HCl, pH 7.5, 56 μM αS
[84]	−2	WT	DPPC/GM3	10:1	20 mM Tris-HCl, pH 7.5, 56 μM αS
[101]	1	WT	WT exosomes	30 μM αS, 0.25 mg/mL exosomes	10 mM MES, 140 mM NaCl, pH 5.5, 30 μM αS,100 rpm
[101]	1	WT	overexpressing exosomes	30 μM αS, 0.25 mg/mL exosomes	10 mM MES, 140 mM NaCl, pH 5.5, 30 μM αS, no shaking
[101]	1	A_53_T	A_53_T exosomes	25:3	10 mM MES, 140 mM NaCl, pH 5.5, 30 μM αS,100 rpm
[101]	1	A_30_P	A_30_P exosomes	25:3	10 mM MES, 140 mM NaCl, pH 5.5, 30 μM αS,100 rpm
[101]	1	E_46_K	E_46_K exosomes	25:3	10 mM MES, 140 mM NaCl, pH 5.5, 30 μM αS,100 rpm
[101]	−1	WT	DOPC	20:3	10 mM MES, 140 mM NaCl, pH 5.5, 30 μM αS,100 rpm
[101]	−1	WT	DOPC 6% Chol	20:3	10 mM MES, 140 mM NaCl, pH 5.5, 30 μM αS,100 rpm
[101]	1	WT	WT exosomes	30 μM αS:.25 mg/mL exosomes	10 mM MES, 140 mM NaCl, pH 5.5, 30 μM αS,100 rpm
[95]	−1	WT	GM1	10:1	50 mM ammonium acetate, pH 7.4, 42 μM αS, shaking
[95]	−2	WT	GM1	10:1	50 mM ammonium acetate, pH 7.4, 42 μM αS, shaking
[94]	1	WT	DOPC/GM1 9:1	5:1, 10:1, 15:1, 20:1	10 mM MES, pH 5.5, 20 μM αS, no shaking
[94]	1	WT	DOPC/GM3 9:1	5:1, 10:1, 15:1, 20:1	10 mM MES, pH 5.5, 20 μM αS, no shaking
[94]	1	WT	DOPC/PE-PEG750 9:1	5:1, 10:1, 15:1, 20:1	10 mM MES, pH 5.5, 20 μM αS, no shaking
[94]	−2	WT	DOPC/Asialo-GM1 9:1	5:1, 10:1, 15:1, 25:1, 50:1	10 mM MES, pH 5.5, 20 μM αS, no shaking
[94]	−2	WT	DOPC/CE-PEG750 9:1	5:1, 10:1, 15:1, 25:1, 50:1	10 mM MES, pH 5.5, 20 μM αS, no shaking
[99]	- -	HNE	DOPE/DOPS/DOPC 5:3:2	9:2, 12:2, 15:2, 20:2	20 mM Tris, pH 7.4, 140 μM αS, no shaking
[100]	0	WT	DOPC/SM/Chol 2:2:1	1:1	20 mM MOPS, 100 mM NaCl, pH 7, 50 μM αS, continuous shaking
[100]	−1	WT	DOPC/SM/Chol 2:2:1	10:1	20 mM MOPS, 100 mM NaCl, pH 7, 50 μM αS, continuous shaking
[88]	1	WT	DOPE/DOPS/DOPC 5:3:2	10:1	20 mM sodium phosphate, 1 mM sodium azide, pH 7.4, 5 μM αS, 180 rpm
[98]	1	WT	DHA	10:1	PBS, pH 7.4, 50 μM αS, 500 rpm
[98]	−2	WT	DHA	50:1	PBS, pH 7.4, 50 μM αS, 500 rpm
[93]	−2	WT	DHA	50:1	20 mM Tris, pH 7.4, 150 mM NaCl, 50 μM αS, 500 rpm

- - For a given protein concentration, the rate decreases as the concentration of lipid increases. ^ Aggregation rates for mutants, T_1/2_(Mut). Mutants are scored by qualitatively comparing the relative rate, T_1/2_(Mut), to the corresponding WT control, T_1/2_(WT), as follows: +1 for T_1/2_(Mut) < 0.5 T_1/2_(WT), 0 for 0.5 T_1/2_(WT) ≤ T_1/2_(Mut) ≤ 2 T_1/2_ (WT), −1 for 2 T_1/2_ (WT) < T_1/2_ (Mut) < 4 T_1/2_ (WT), −2 for T_1/2_ (Mut) > 2 T_1/2_ (WT) or very shallow, non-sigmoidal aggregation curve.

## 3. Discussion

The aggregation of αS plays a crucial role in the advancement of synucleinopathies. Studies have demonstrated that αS fibrils, oligomers, and LB formation are toxic to cells. Consequently, interventions that inhibit aggregation or facilitate the degradation of toxic aggregates could be valuable in treating synucleinopathies. αS’s interactions with lipids also play a role in modulating its aggregation based on the concentration and composition of the lipid. Since one function of WT αS is vesicle trafficking [102], accompanied with the fact that lipids are a major component of LBs [76], these interactions are important for understanding the transformation from functional αS into aggregation-prone αS.

**Figure 5:**
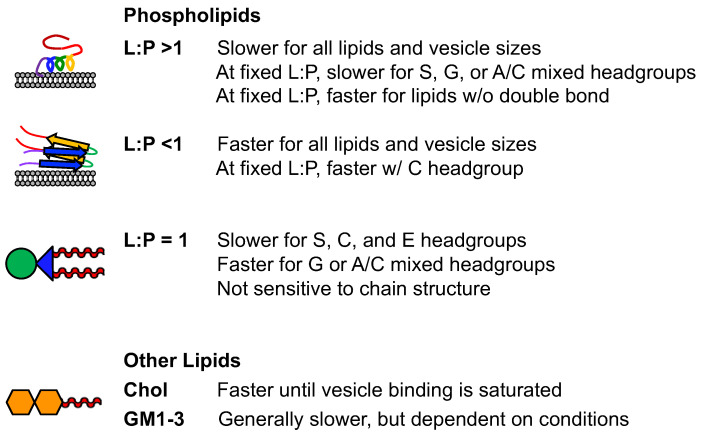
Summary of lipid effects on aggregation. See Table 1 and Figure 4 for abbreviations and structures.

The impact of phospholipids on αS aggregation varies depending on the type of lipid and its concentration relative to αS. Certain lipids with specific L:P ratios can accelerate fibril formation [74] by providing nucleation sites and enabling elongation. However, when the L:P ratio exceeds the number of lipid-bound αS molecules, aggregation is inhibited, as the helical form of membrane-bound αS hinders fibril formation [91]. Lipids with negatively charged headgroups, such as PG [93], have been observed to inhibit aggregation, while zwitterionic PC lipids show mixed effects [83,85]. Lipids that can be remodeled by αS tend to inhibit aggregation by reducing the available free αS for fibril formation. Different lipid lengths and solubilities can also modulate the rate of aggregation. These effects are summarized in Figure 5, where we present the findings in three L:P ratio regimes. This ratio tends to be the most important characteristic, and structural trends can then be interpreted at fixed L:P ratios within that regime.

The effect of gangliosides on αS aggregation is subject to differing views. Some studies have indicated that certain gangliosides, like GM1, can inhibit aggregation [84], while others have suggested that gangliosides, similar to GM1, accelerate fibril formation [94]. Additionally, the results may vary depending on the L:P ratio [94] and the specific conditions of the aggregation assay, such as pH [96] and agitation. In the context of fatty acids, DHA levels change with age, and it can either speed up or inhibit αS fibril formation, affecting aggregation outcomes. The interaction of αS with biologically relevant membranes is important for its proposed functions. Synthetic vesicles mimicking lipid rafts have shown mixed effects on aggregation, and cholesterol content influences αS binding to vesicles and aggregation rates. Exosomes from mouse neuroblastoma cells overexpressing various forms of αS can accelerate fibril formation. The mimics of these exosomes, with different lipid compositions, had mixed effects on aggregation rates, and the impact of GM1 and GM3 vesicles varied with their concentration. However, in vitro vesicles have limitations as models of biologically relevant vesicles. Direct interactions between lipids and fibrils, resulting from lipid-associated aggregation, could offer the structural foundation for a lipid extraction mechanism [24]. Indeed, structures of lipid-coated fibrils have recently been reported, providing the first insight into how lipid molecules can influence fibrils [103].

**Figure 6:**
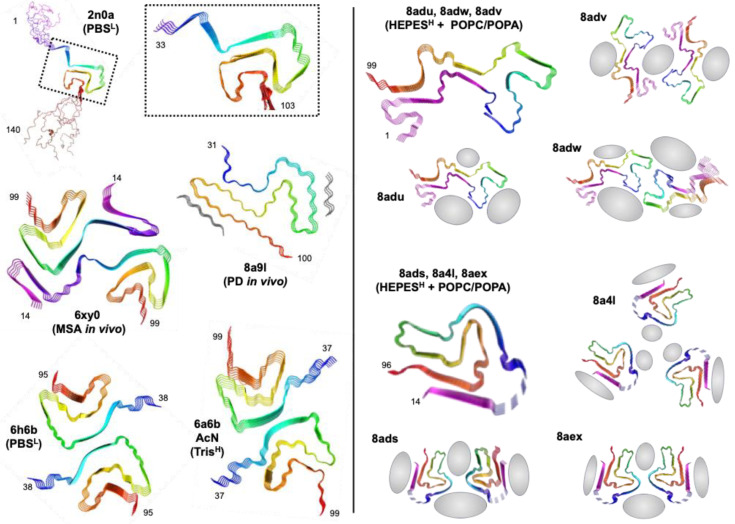
Lipid effects on αS fibril structures. Fibril structures from ssNMR (2n0a) and cryo-EM (all other PDB IDs) with aggregation conditions (buffer and salt; low = L or high = H). Two fibril conformers are shown for lipid-bound fibrils, each with three different strand packing orientations. Regions of lipid interactions identified from cryo-EM, ssNMR, and MD are shown in gray.

Frieg et al. performed αS aggregations in the presence of 1.5 mM SUVs composed of 1:1 POPA/POPC at 5:1 L:P, and observed the resulting fibrils through cryo-electron microscopy (cryo-EM) as well as solid-state NMR (ssNMR) [103]. Two protofibril polymorphs were observed, with three different packing orientations for each type of protofibril (Figure 6). Neither polymorph resembled previously reported polymorphs, including in vitro fibril structures of αS [60,104,105] and those obtained for fibrils isolated from MSA [106] or PDD [107] patients. Since it had previously been shown that aggregation conditions impacted fibril polymorphism, it is important to note that their fibrils were formed in 50 mM HEPES, 100 mM NaCl, and pH 7.4, whereas previously reported fibril structures used phosphate-buffered saline (PBS), with low (10 mM) or high (100 mM) salt, or Tris buffer with high salt. Thus, while the influence of buffer cannot be completely excluded, it is unlikely to be responsible for the drastically different fibril folds that they observed, since PBS and Tris folds are generally similar. Moreover, they were able to directly observe a well-resolved density for some lipids bound to the fibril surface, as well as an additional density that they assigned to specific functional group interactions based on ssNMR data as and molecular dynamics (MD) simulations. While these images only represent one class of lipid interactions, for a particular composition, L:P, and vesicle size, they represent important insights and already show just how dramatically αS fibrils can be altered by the presence of lipids, showing that lipid interactions will not only alter aggregation by changing nucleation steps, but also by altering fibril end products.

## 4. Conclusions

Despite the limitations in accounting for varying aggregation conditions, studying the effects of lipids on αS aggregation is crucial due to the central role αS plays in neurodegenerative diseases. Lipids, particularly those found in biologically relevant membranes like lipid rafts, vesicles, and exosomes, can modulate αS aggregation rates. Understanding how different lipid compositions impact αS aggregation can shed light on disease mechanisms and potential therapeutic strategies. The interactions between αS and lipids offer valuable insights into the underlying molecular processes involved in synucleinopathies, providing opportunities for targeted interventions to prevent or treat these debilitating conditions.

## Figures and Tables

**Table 1:** Lipid names and abbreviations.

Full Name	Abbreviation
Cerebroside	CB
Ceramide	CE
Cholesterol	Chol
1,2-Dilauroyl-*sn*-glycero-3-phospho-L-serine	DLPS
1,2-Dimyristoyl-*sn*-glycero-3-phospho-L-serine	DMPS
1,2-Dioleoyl-*sn*-glycero-3-phospho-L-serine	DOPS
1,2-Dipalmitoyl-*sn*-glycero-3-phosphate	DPPA
1,2-Dipalmitoyl-*sn*-glycero-3-phosphocholine	DPPC
1,2-Dipalmitoyl-*sn*-glycero-3-phosphoglycerol	DPPG
1,2-Dipalmitoyl-*sn*-glycero-3-phosphoserine	DPPS
Ganglioside GM1	GM1
Ganglioside GM2	GM3
Ganglioside GM3	GM3
4-Hydroxynonenal	HNE
1-Palmitoyl-2-oleoyl-*sn*-glycero-3-phosphate	POPA
1-Palmitoyl-2-oleoyl-*sn*-glycero-3-phosphocholine	POPC
1-Palmitoyl-2-oleoyl-*sn*-glycero-3-phosphoglycerol	POPG
1-Palmitoyl-2-oleoyl-sn-glycero-3-phosphoinositol	POPI
1-Palmitoyl-2-oleoyl-*sn*-glycero-3-phospho-L-serine	POPS
Sialic acid	SA
Sphingomyelin	SM
Sphingosine	SP

## Data Availability

Spreadsheets containing the original data analysis used to prepare Table 2 and Table 3 can be made available by email to the corresponding author.
